# The β-adrenergic receptor antagonist propranolol offsets resistance mechanisms to chemotherapeutics in diverse sarcoma subtypes: a pilot study

**DOI:** 10.1038/s41598-020-67342-6

**Published:** 2020-06-26

**Authors:** Letizia Porcelli, Marianna Garofoli, Roberta Di Fonte, Livia Fucci, Mariateresa Volpicella, Sabino Strippoli, Michele Guida, Amalia Azzariti

**Affiliations:** 1 Experimental Pharmacology Laboratory, IRCCS Istituto Tumori Giovanni Paolo II, Viale O. Flacco, 65, 70124 Bari, Italy; 2 Department of Biosciences, Biotechnologies and Biopharmaceutics, University of Bari, Bari, Italy; 3 Histopathological Unit, IRCCS Istituto Tumori Giovanni Paolo II, Bari, Italy; 4 Medical Oncology, IRCCS Istituto Tumori Giovanni Paolo II, Bari, Italy

**Keywords:** Cancer therapy, Sarcoma

## Abstract

Standard chemotherapy for soft tissue sarcomas has shown limited efficacy. Here, we sought to evaluate whether β-adrenergic receptor (β-AR) signalling contributed to the progression of sarcomas and therapy resistance. To assess the translational potential of β-adrenergic receptors, we performed immunohistochemical detection of β1-AR, β2-AR and β3-AR in leiomyosarcoma, liposarcoma and angiosarcoma tissue specimens, reporting the results scored for the intensity. By using established and patient-derived sarcoma cells, we demonstrated the antitumour potential of the pharmacological targeting of β-ARs with the nonselective β-blocker propranolol in such sarcomas. Of note, pharmacological β-AR inhibition synergized with doxorubicin in inhibiting the cell viability of liposarcoma and leiomyosarcoma cells and increased the response to docetaxel in angiosarcoma- and solitary fibrous tumour (SFT)-patient-derived cells. Notably, the SFT patient was treated with the combination of propranolol and docetaxel, reporting prolonged disease control. Mechanistically, we found that propranolol reduced the activity of the multidrug resistance efflux pump P-gp, thereby increasing the intracellular doxorubicin concentration and antitumour activity. In addition, propranolol attenuated the Akt-dependent survival signal induced by doxorubicin and strongly reduced the activation of the NF-kB/COX-2 pathway, increasing cell sensitivity to docetaxel. Overall, our study highlighted the therapeutic potential of propranolol, alone or in rational combination therapies, for sarcoma treatment.

## Introduction

Soft tissue sarcomas (STSs) are a heterogeneous group of mesenchymal cancers that account for more than 60 histological types with specific characteristics, such as biological behaviour, natural history and treatment responsiveness^1^. However, despite the heterogeneous histology, available therapeutic options rely on doxorubicin-based chemotherapy regimens, though in the advanced stage, this has invariably led to a dismal prognosis of STS patients^2–4^, with a reported median overall survival shorter than 1 year^5^.

Among the experimental approaches that are being developed to improve patient response to therapy, combinatorial strategies with new targeted therapy have been shown to enhance the efficacy of chemotherapeutics in several clinical and preclinical models of STS^6–10^. From this perspective, in recent years, growing attention has been given to the crucial role of adrenergic processes, originating from adrenergic receptors (ARs), in driving the development of tumours and metastasis^11–13^, since ARs are expressed across diverse cancers^14^. ARs are a class of G protein-coupled receptors (GPCRs) including α and β-types^15^ that, by coupling with catecholamines (norepinephrine, epinephrine), stimulate the formation of intracellular cyclic adenosine monophosphate (cAMP), the second messenger that activates numerous PKA-dependent and independent intracellular signalling cascades^16,17^, promoting the proliferation, metastasis and angiogenesis of numerous cancers^18,19^. Variable expression of β-ARs has been demonstrated in a broad spectrum of human vascular lesions, including angiosarcoma^20,21^, and this has led to the experimentation of the pharmacological inhibition of beta adrenergic receptors in angiosarcoma treatment, reporting increased progression-free and overall survival in such patients^14^. Regarding other sarcoma subtypes, to the best of our knowledge, there are no data on the immunohistochemical expression of β-ARs. Therefore, here, for the first time, we determined the expression of β-ARs in formalin-fixed paraffin-embedded (FFPE) tissue specimens from liposarcoma (LPS) and leiomyosarcoma (LMS) patients. Moreover, because the expression does not indicate that these sarcomas are responsive to β-receptor antagonists, as a proof of concept, we explored whether the β-AR antagonist propranolol resulted in therapeutic efficacy and an enhanced response to standard chemotherapeutics in cell models of LMS and LPS by investigating putative mechanisms underlying the effectiveness of combinations. Focusing on angiosarcoma, because therapeutic advancements are very slow and alternative drugs and cellular models of this disease are urgently needed, we isolated a new angiosarcoma cell line from the biopsy of an angiosarcoma (AS) patient that we utilized to evaluate the expression of β-ARs and to test the antitumour potential of propranolol alone and in combination with docetaxel. We discovered that targeting β-ARs by propranolol inhibited NF-kB/Cox-2 signalling, which is activated in response to docetaxel in AS cells.

More importantly, owing to the vascular ontogeny and the likeness of solitary fibrous tumour (SFT) with angiosarcoma, we explored the incorporation of propranolol in the standard treatment of an SFT patient by testing this therapeutic strategy on a short-term culture obtained from a tumour biopsy of the SFT patient. For this purpose, we determined the expression of β-ARs and evaluated either the effect of propranolol or its combination with docetaxel in patient-derived cells. We found that propranolol significantly enhanced the response to docetaxel in vitro; therefore, the combination was prescribed to the patient, who reported prolonged disease control.

## Results

### IHC expression of β-ARs in AS, LMS and LPS tissue specimens

**Figure 1:**
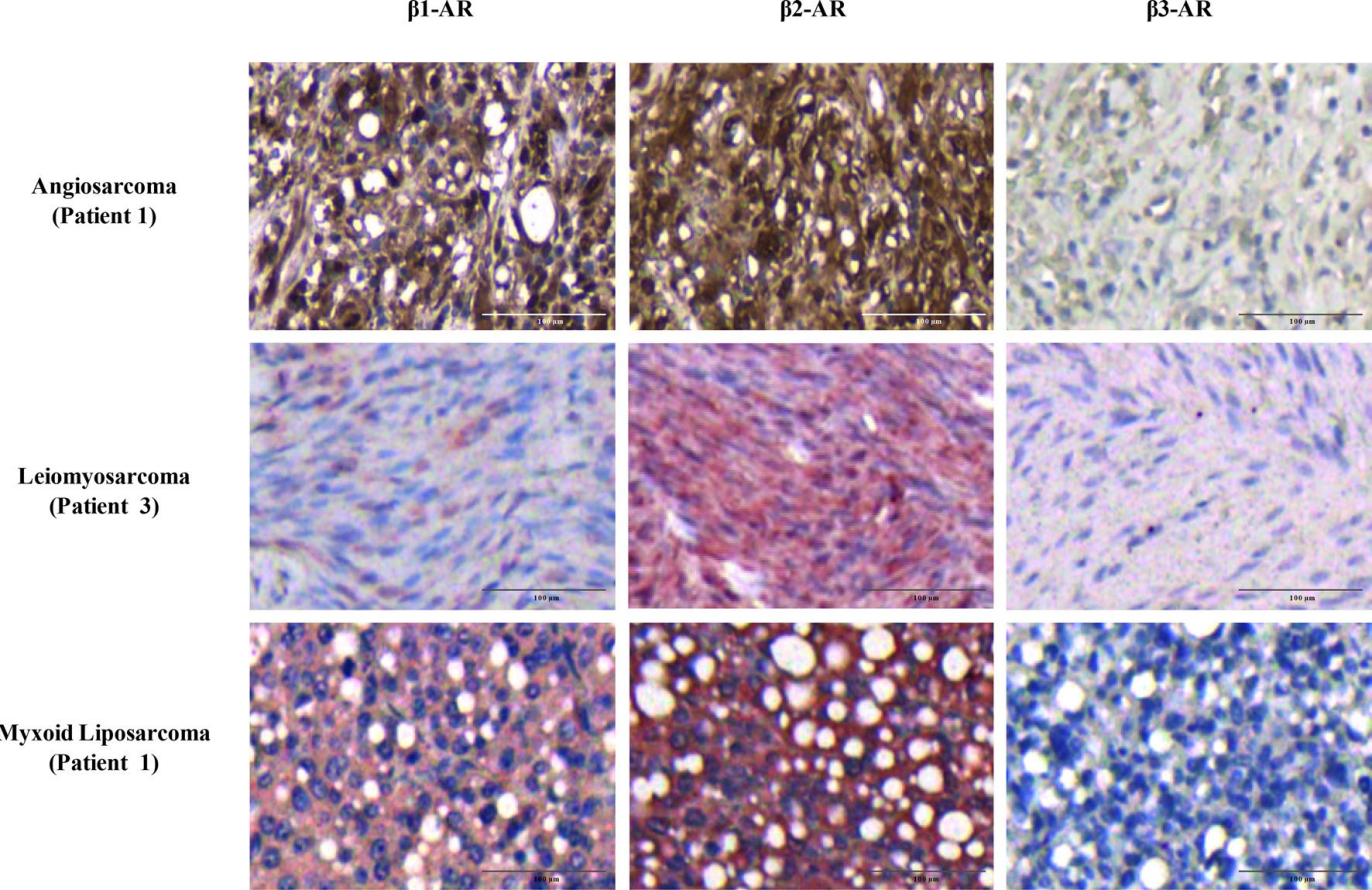
β-Adrenergic receptor (AR) staining in sarcoma specimens. Immunohistochemical evaluation of β1-AR, β2-AR, and β3-AR in angiosarcoma, leiomyosarcoma and myxoid liposarcoma specimens. As shown, β1-AR and β2-AR had moderate and strong expression, respectively, in all sarcoma subtypes, whereas β3-AR was undetectable. Image scale bar, 100 µm.

The FFPE tissue specimens of one AS and of five LPS and LMS patients were immunostained for β1-AR, β2-AR, and β3-AR expression determination. According to the intensity of the staining, the expression level was scored with a four-tiered scale as summarized in Table 1, in which the results of IHC analysis are shown. Representative images from each tumour type are shown in Fig. 1. The AS specimens displayed a moderate expression of β1 and strong expression of β2-AR, in agreement with the IHC evaluation performed by other authors^20^ in vascular tumours, including angiosarcoma. All LMS and LPS specimens showed weak or moderate staining for β1, strong staining for β2 and undetectable staining for β3. Overall, the IHC analysis showed that β-ARs were localized both at the cytoplasmic and nuclear levels. In LMS specimens, the staining was mainly localized in the cytoplasm; instead, most LPS cases and AS specimens showed both nuclear and cytoplasmic expression of β-ARs. Although the IHC evaluation of β-ARs was performed in a small cohort of LPS and LMS patients, the data collected confirmed that β-ARs are expressed across different sarcoma subtypes. The images collected from all analyses are provided in Supplementary Figures S1 and S2.

**Table 1:** Intensity score of β-ARs in AS, LMS and LPS tissue specimens by IHC.

Histology	N. cases	Patients	Original location	Grade	β1-AR	β2-AR	β3-AR
Angiosarcoma	1	1	Scalp	Primitive	++	+++	–
Leiomyosarcoma	5	1	Jejunum	Metastasis	+	+++	–
		2	Uterus	Primitive	++	+++	–
		3	Skin	Primitive	+	+++	–
		4	Pelvis	Relapse	+	+++	–
		5	Skin	Metastasis	++	+++	–
Dedifferentiated liposarcoma	3	1	Retroperitoneal	Primitive	++	+++	–
		2	Thigh	Relapse	+	+++	–
		3	Breast	Primitive	++	+++	–
Atypical lipomatous tumor	1	1	Retroperitoneal	Primitive	+	+++	–
Myxoid liposarcoma	1	1	Retroperitoneal	Primitive	++	+++	–

The quantification of β-AR expression in AS, LMS and LPS tissue specimens was evaluated using the following criteria: – undetectable staining; + weak; ++ moderate; +++ strong.

### Establishment and characterization of a patient-derived angiosarcoma cell line, designated AS-O2

**Figure 2:**
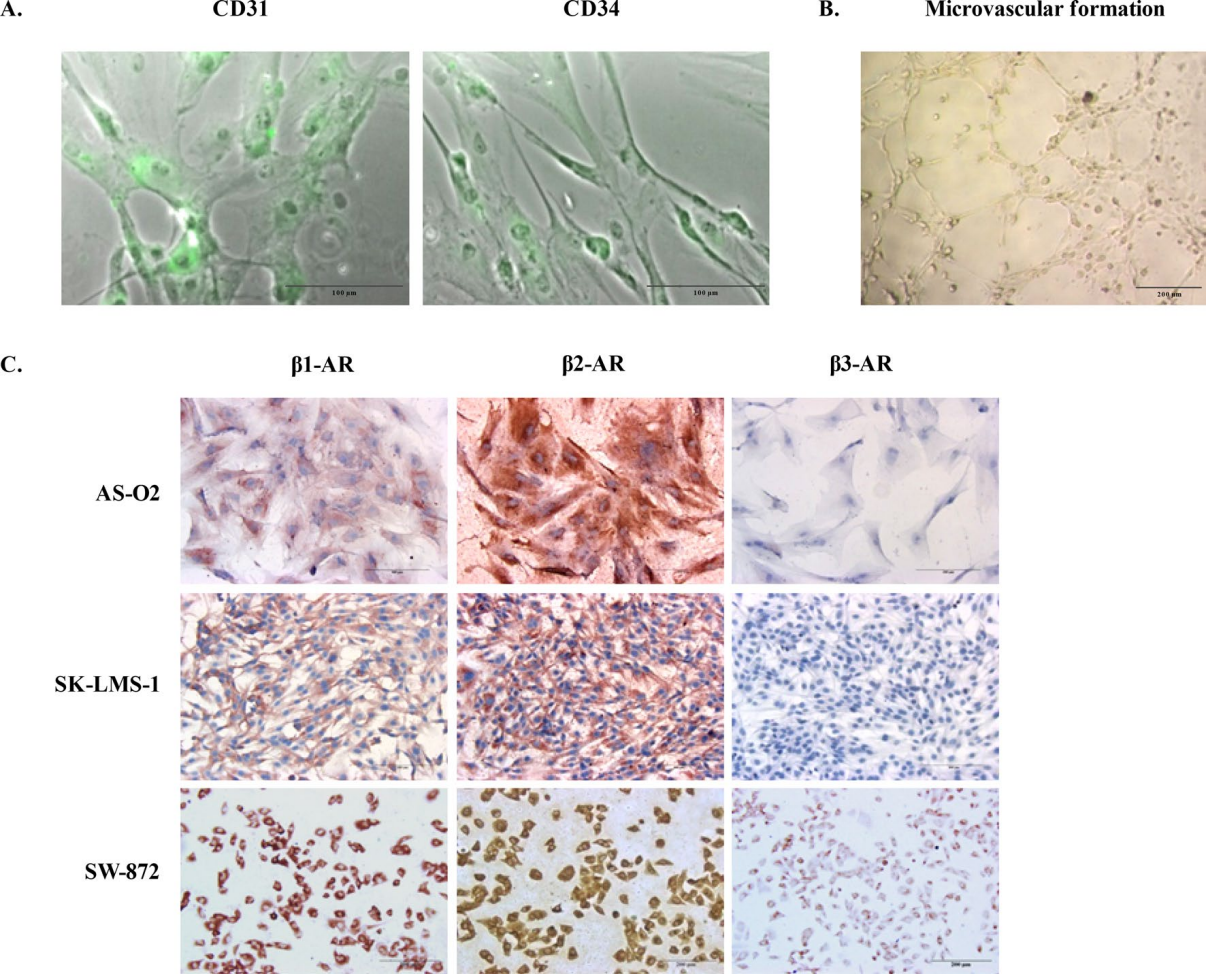
Characterization by immunostaining and microvascular formation assay on Matrigel of patient-derived angiosarcoma cells designated AS-O2 and β-AR expression in all STS cells. (**A**) Representative images of fluorescence and phase-contrast microscopy captured from AS-O2 cells showing the expression of both CD31 and CD34 (green fluorescence). Image scale bar, 100 µm. (**B**) Representative image showing the capability of AS-O2 cells to form microvascular structures. Image scale bar, 200 µm. (**C**) Representative images reporting β1-AR, β2-AR and β3-AR in AS, LMS and LPS cell lines, evaluated by ICC. Image scale bar, 200 µm.

Cell lines are a valuable tool for the preclinical evaluation of anticancer drugs; however, there is a lack of patient-derived cells of angiosarcoma. For this purpose, a primary cell line designated AS-O2 was established from a lesion of the scalp of a 79-year-old male patient with a diagnosis of angiosarcoma. Of note, he was the same patient who showed IHC expression of β-ARs as reported in Table 1. As reported in our previous report^22^, we characterized the cells at an early passage by analysing the expression of CD31 and CD34 to confirm that they were representative of the tumour of origin. According to the nature of vascular tumours, they were positive for CD31 and CD34 staining (Fig. 2A). In addition, the cells organized with the characteristic endothelial pattern of microvascular formation once seeded on Matrigel (Fig. 2B).

### Propranolol inhibits proliferation and increases the effectiveness of chemotherapy in AS, LMS and LPS cell lines expressing β-adrenergic receptors

**Figure 3:**
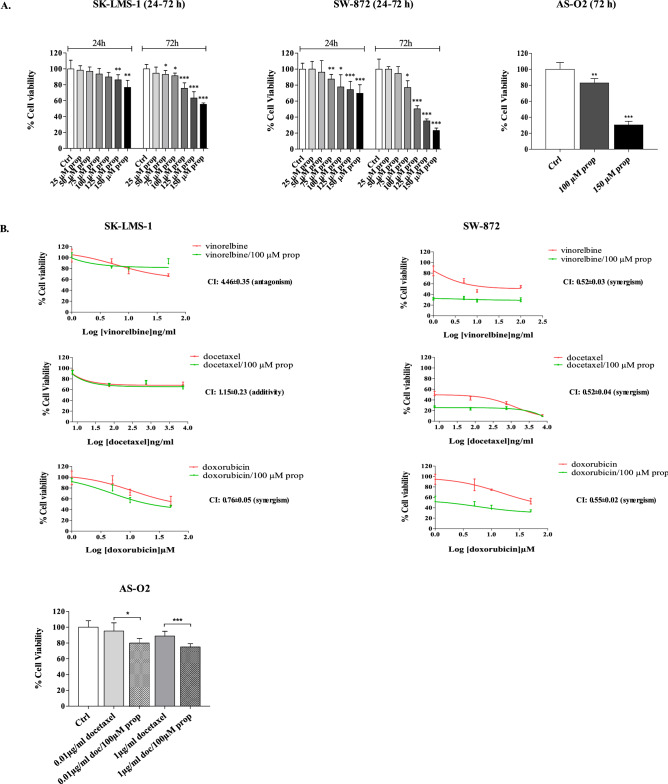
Therapeutic efficacy of propranolol alone and in combination with chemotherapeutics in sarcoma cells expressing β-ARs. (**A**) SK-LMS-1 and SW-872 cells were incubated with propranolol (prop) ranging from 25 to 150 µM for 24–72 h. AS cells were incubated with 100 and 150 µM propranolol for 72 h. Histogram plots report the inhibition of cell viability as the mean value ± SD of three independent experiments (***p < 0.001; **p < 0.01; *p < 0.5 were calculated versus untreated cells). (**B**) Dose–response plots and CI showing the inhibition of cell viability and pharmacological interaction of drugs in SK-LMS-1, SW-872 and AS-O2 cells.

A possible role of β-ARs as pharmacological targets in AS-O2, SK-LMS-1 and SW-872 cell lines was investigated. The expression of β-AR types 1, 2 and 3 was determined by ICC as displayed in Fig. 2C. β-AR types 1 and 2 were expressed, even with different intensities, in all cells, confirming the results found in LPS and LMS tissue samples reported in Table 1. The LPS cells (SW-872) were positive for only β3-AR expression (Fig. 2C). Cell sensitivity to propranolol was determined as a function of drug concentration and time exposure. Unlike LPS and LMS cells, AS cells were challenged with only two concentrations of propranolol, i.e., 100 and 150 µM because, as reported by others^23^, such tumour cells displayed a very low rate of cell division in vitro, which limits the availability of a suitable number of replicates in each series of experiments. In all cell lines, prolonged exposure to propranolol (72 h vs. 24 h) resulted in an increased antitumour effect. The AS and LPS cells were the most sensitive to β-AR antagonists, since at the highest concentration of propranolol, the cell viability of both was reduced by approximately 70% compared to 40% in LMS cells. The results of the cytotoxicity study are reported in Fig. 3A. Because β-ARs trigger signaling networks that notoriously drive resistance to standard chemotherapy, we tested the hypothesis of combining propranolol with some chemotherapeutics included in the standard treatment of STSs (i.e. doxorubicin, docetaxel and vinorelbine) to enhance the response to treatment. To perform the combination study, we chose 100 µM as the effective dose (ED) of propranolol after 24 h, which corresponded to the ED80 in almost all cell lines and to the range of concentrations utilized in several reports evaluating the antitumour effect of propranolol in vitro^24,25^. We tested the combination with doxorubicin, docetaxel and vinorelbine in LPS and LMS cells and only with docetaxel in AS cells because taxanes are an established treatment in this sarcoma subtype, as demonstrated by a phase II study^26–28^. The effects on cell viability are summarized in Fig. 3B, in which the dose–response plots of the combinations and of each chemotherapeutic drug are shown. The combination index (CI) was calculated to evaluate the pharmacological interaction between propranolol and each chemotherapeutic drug when the combination inhibited 50% of cell viability. As shown by the growth inhibition curves and CIs (Fig. 3B), all the combinations had a greater effect than the chemotherapeutics alone in LPS cells. Only the combination with doxorubicin was more effective than chemotherapeutic alone, even in LMS cells. In AS cells, the addition of propranolol resulted in an increase of 20% in the response to a subtoxic dose of docetaxel (0.01 µg/ml) and of 27% of the response to 1 µg/ml docetaxel (Fig. 3B).

### Propranolol offset resistance mechanisms to doxorubicin in LMS and LPS cells

**Figure 4:**
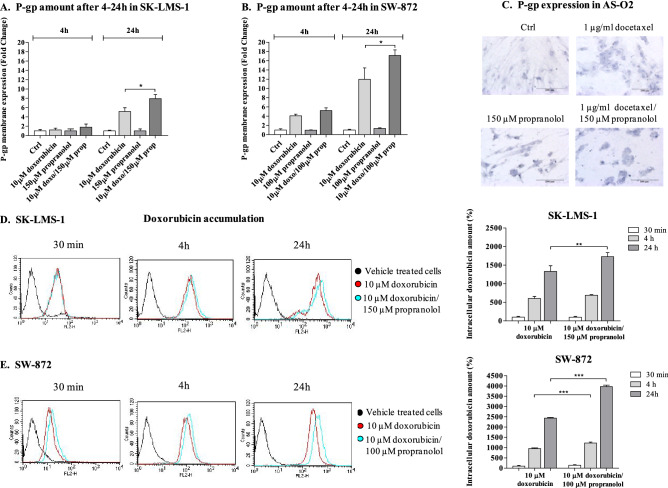
P-gp expression evaluation and intracellular accumulation of doxorubicin in LMS, LPS and AS cells. (**A**,**B**) Histogram plots reporting the P-gp expression on the plasma membrane of SK-LMS-1 and SW-872 cells after doxorubicin, propranolol and drug combination, evaluated at 4 and 24 h by FACS analysis. (**C**) Evaluation of P-gp expression by ICC on AS cells after incubation with docetaxel, propranolol and drug combinations. Image scale bar, 200 µm. (**D**,**E**) Representative images showing the intracellular doxorubicin content in SK-LMS-1 and SW-872 cells, respectively, performed by FACS after 30 min, 4 and 24 h of treatment with doxorubicin, propranolol and drug combinations. Histogram plots report the intracellular content of doxorubicin, calculated as the mean ± SD of three independent experiments (***p < 0.001; **p < 0.01; *p < 0.05; calculated with respect to doxorubicin given alone).

Focusing on the mechanisms underlying the effectiveness of propranolol in combination with docetaxel or doxorubicin, we investigated the hypothesis that propranolol might impact chemotherapeutic efficacy by modulating P-glycoprotein (P-gp) cellular expression and activity. P-gp plays a pivotal role in regulating the intracellular accumulation and retention of both doxorubicin and docetaxel and is crucial in conveying a multidrug resistance phenotype^29,30^ to several tumours. For this purpose, we evaluated the expression level of P-gp on the cell membrane of SK-LMS-1 and SW-872 cells during drug treatment by flow cytometry (FCM), and because doxorubicin has fluorescent properties, we determined the accumulation of the drug in cells by FCM. P-gp expression was instead determined by ICC on AS-O2 cells. At time zero, both SK-LMS-1 and SW-872 cells showed slight positivity for P-gp expression on the plasma membrane (data not shown). After 4 h/24 h, propranolol did not alter the expression of P-gp on the plasma membrane; instead, doxorubicin induced a time-dependent increase that was stronger in SW-872 cells than in SK-LMS-1 cells (eightfold vs. fourfold). The drug combination further increased the expression of P-gp after 24 h in both cell lines, and again, the extent of the increase was greater in SW-872 cells than in SK-LMS-1 cells (12-fold versus sixfold). These data are summarized in histogram plots in Fig. 4 A/B. AS-O2 cells were negative for the expression of P-gp, as displayed by ICC analysis, and neither the addition of docetaxel nor propranolol induced a detectable level of P-gp expression by ICC, as displayed by representative images shown in Fig. 4C. Nonetheless, the combination significantly augmented the intracellular content of doxorubicin compared to doxorubicin alone in both the SK-LMS-1 and SW-872 lines. The intracellular amount of doxorubicin was evaluated by FCM as a function of treatment and time. In SK-LMS-1 cells, by fixing at 100% doxorubicin accumulation after 30 min, we found that it reached 1,300% after 24 h, and notably, when the cells were incubated with the drug combination, the increase in doxorubicin at 24 h was 1,700% (Fig. 4D). In SW-872 cells, by fixing the accumulation of doxorubicin at 100% after 30 min, we found an increase of 2,400% at 24 h and a further increase to 4,000% in the combination with propranolol (Fig. 4E). This demonstrated, in agreement with the findings of Bachmakov et al.^31^, that propranolol inhibited the P-gp-dependent transport of doxorubicin in both LPS and LMS cells and that the higher the expression of P-gp is on the membrane, the stronger the inhibition of doxorubicin efflux by propranolol.

**Figure 5:**
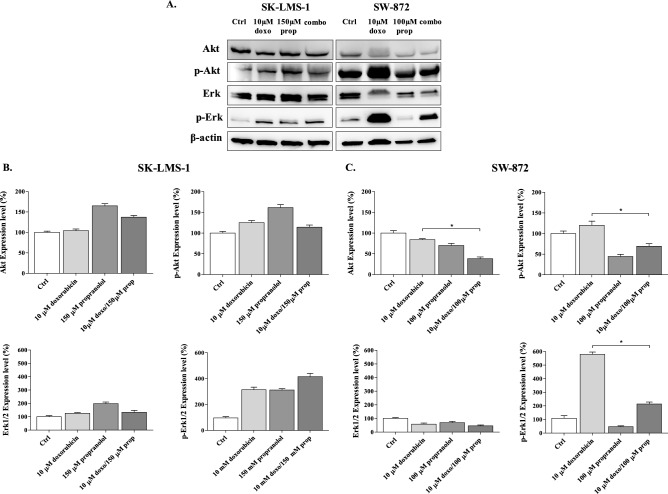
Effects of drugs on the expression levels of p-Akt, Akt, p-Erk1/2, Erk1/2. (**A**) Immunoblots performed on protein extracts from SK-LMS-1 and SW-872 cells treated with doxorubicin, propranolol and their combinations are reported. (**B**, **C**) Bar graphs show quantification by densitometric analysis of protein bands from two independent experiments (*p < 0.05).

Both PI3K/Akt and MAPK/Erk1/2 signalling are associated with the mechanisms of resistance to chemotherapy^32,33^ in multiple cancer cells^34–36^ because they are involved in the regulation of expression and activity of relevant MDR proteins^37^, such as P-gp. As reported above, β-ARs trigger such signalling pathways to promote tumour proliferation; hence, we determined the effects of drugs on the expression and activation status of Erk1/2 and Akt to find a correlation with the tumour cell response to doxorubicin (Fig. 5A, Figure S3).

This evaluation revealed that in LMS cells, doxorubicin significantly increased the phosphorylation of both Akt and Erk1/2, whereas propranolol augmented only the activation status of Erk (Fig. 5A). In combination, propranolol lowered the doxorubicin-dependent activation of Akt while further increasing the activation of Erk1/2 (Fig. 5A). In LPS cells, both doxorubicin and propranolol lowered Akt-dependent survival signalling either as a single agent or in combination (Fig. 5A). Doxorubicin alone increased p-Erk1/2, while in combination, propranolol strongly reduced the doxorubicin-dependent activation of Erk1/2 (Fig. 5A). The quantification results are summarized in Fig. 5B, C. Overall, these data suggest that the constitutive activation of β-ARs may modulate cell proliferation and the response to doxorubicin preferentially through the modulation of Akt signalling in SK-LMS-1 cells and through both Akt and Erk1/2 signalling in SW-872 cells. Perhaps in both LMS and LPS cells, p-Erk was involved in resistance mechanisms to chemotherapy, such as sustaining the expression of P-gp on the membrane, while p-Akt was mainly involved in the activation of survival signalling adopted by cells to counteract therapy.

### Impact of COX-2 expression on the response to docetaxel in angiosarcoma cells

**Figure 6:**
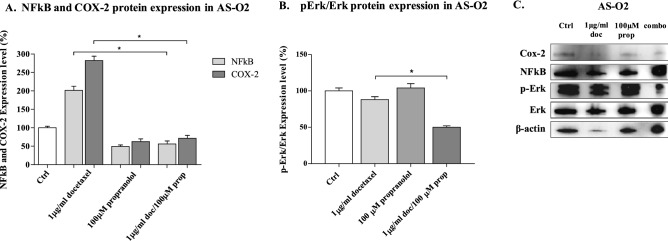
Western Blot analysis of protein extracts from AS cells treated with docetaxel and propranolol alone and in combination. The bar graph shows quantification by densitometric analysis of (**A**) NF-kB, COX-2 and (**B**) p-Erk1/2/Erk1/2 protein bands from two independent experiments (*p < 0.05). (**C**) Representative Western blot protein bands.

Both Cox-2 and NF-kB expression/activity have been linked to resistance to doxorubicin and taxane-based chemotherapy in several tumour types^38,39^. Because AS-O2 cells are slightly sensitive to docetaxel and exhibit constitutive activation of β-AR signalling that notoriously triggers pro-survival signals through NF-κB and Cox-2 signalling in several cancer types^17^, we hypothesized that propranolol enhanced the sensitivity of cells to docetaxel through the inhibition of the β-AR/NF-κB-COX-2 pathway. For this purpose, we performed Western blot analysis on cell lysates from cells treated with each drug and their combination to determine the effects on NF-kB and Cox-2 expression (Fig. 6A–C). We found that docetaxel strongly stimulated the expression of both NF-kB and Cox-2 compared with that in untreated cells. Propranolol instead strongly lowered the baseline expression of NF-kB and Cox-2, and in combination, it completely abrogated the docetaxel-induced stimulation of both biomarkers. Following treatment with the β-blocker or docetaxel, no inhibition of p-Erk1/2/Erk1/2 was observed; instead, with the drug combination, the p-Erk1/2/Erk1/2 ratio was reduced by approximately 50% compared that in untreated cells (Fig. 6B,C), as shown by the significant inhibition of angiosarcoma cell proliferation.

### Cytotoxicity of propranolol and chemotherapeutics in the SFT model and clinical efficacy of propranolol in patients with SFT

**Figure 7:**
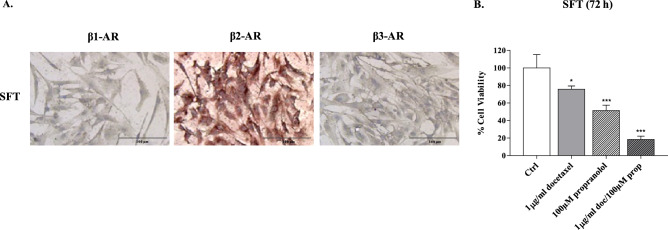
Immunocytochemical staining and propranolol plus docetaxel effectiveness in SFT primary cell models. (**A**) β-AR expression in SFT cells. Image scale bar, 100 µm. (**B**) SFT cells were incubated with docetaxel alone or in combination with propranolol for 72 h. The inhibition of cell proliferation, shown in the histogram, is the mean median of three experiments (mean ± SD, ***p < 0.001; *p < 0.05; where* p* values of single drugs were calculated with respect to Ctrl, while the *p* value of combination was calculated with respect to single drugs).

To broaden the evaluation of β-ARs as tumour targets in diverse human sarcomas, we prepared a short-term culture from the biopsy of an SFT male patient to determine β-AR expression and sensitivity to the β-adrenergic receptor antagonist propranolol. The results of ICC analysis showed that β-AR type 2 was strongly expressed by cells of this patient who was being treated with docetaxel as third line therapy (Fig. 7A). Therefore, we determined the cytotoxicity of docetaxel in combination with propranolol. We found that 100 µM propranolol yielded 50% inhibition of cell viability, showing that constitutive activation of β-AR2 drives the proliferation of such cells, and of note, in combination with docetaxel, it strongly reduced cell viability from 24 to 82% (docetaxel vs. combination) (Fig. 7B).

Propranolol was used in combination with docetaxel in a 50-year-old male patient from whom the SFT cell line was established. In this patient, SFT was diagnosed in May 2012 with a biopsy of a soft tissue enlarging mass on the right shoulder. At first staging, a CT scan showed liver and soft tissue involvement; thus, the patient commenced first-line therapy with doxorubicin and ifosfamide for 4 cycles. Seven months later, the patient experienced disease progression and was treated with second-line therapy with pazopanib. He obtained stable disease as the best response with a progression-free survival of 39 months. In July 2017, as the hepatic lesions increased, the patient started docetaxel (50 mg/m^2^ biweekly) plus propranolol (40 mg daily) therapy. With this therapeutic strategy, the patient showed a prolonged stable disease of 18 months that represented an unexpected outcome for third-line therapy. The treatment has been well tolerated except for occasional hypoglycaemia that has been controlled with symptomatic therapy. After progression, the patient began a fourth-line therapy with gemcitabine, but after 2 months, in February 2019, he died because of further liver progression of the disease.

## Discussion

To the best of our knowledge, this is the first report demonstrating that β-ARs are invariably expressed in different types of human sarcomas and provides a rationale for the utilization of the non-selective β-AR antagonist propranolol to enhance the response to standard chemotherapy. The main findings of the present study are as follows: (1) β-ARs are expressed and might be tumour targets in human LPS and LMS; (2) the immunohistochemical expression of β-ARs in specimens of angiosarcoma patients might predict the response to propranolol in combination with docetaxel-based chemotherapy; and (3) the short-term culture of patient-derived SFT cells expressing β-ARs displayed sensitivity to propranolol in combination with docetaxel, which was reflected in the patient clinical outcome. Following the suggestion of Lee and co-authors, who demonstrated that β-ARs are expressed and have an important functional role in the benign counterparts of LMS and LPS (i.e., leiomyoma and lipoma^19^), we found that β-AR signalling is involved in the proliferation of liposarcoma and leiomyosarcoma cells, since the β-AR antagonist propranolol effectively inhibited cell proliferation, though to a higher extent in SW-872 liposarcoma than leiomyosarcoma cells. Indeed, the combination of propranolol with all tested chemotherapeutics achieved a synergistic inhibition of proliferation in SW-872 liposarcoma cells, further suggesting a functional role of β-AR signalling in proliferation and in the response to chemotherapeutics in these cells. In addition, propranolol offset mechanisms of resistance to doxorubicin in SW-872 cells, as it attenuated the Akt-dependent survival signal, which is regarded as a prognostic factor for STSs^40^ and a key player in the resistance to chemotherapeutics in several tumour types^41^. Along with the inhibition of Akt signalling, propranolol reduced the expression and activation of Erk1/2 induced by doxorubicin in liposarcoma cells. Although the doxorubicin-induced activation of Erk1/2 has been regarded as a mechanism of drug effectiveness in several tumour types^42^, it had never been investigated in liposarcoma; therefore, further investigation is warranted. Of note, in both liposarcoma and leiomyosarcoma, the addition of propranolol to doxorubicin further increased the expression of P-gp on the plasma membrane; nonetheless, as reported by other authors^37^, it strongly inhibited the P-gp-mediated efflux of doxorubicin from the cells, thus resulting in an increase in the intracellular retention and effectiveness of doxorubicin^31^. Focusing on angiosarcoma, we excluded that propranolol increased the response to docetaxel by inhibiting the P-gp-mediated transport of the chemotherapeutic agent; instead, our findings highlighted the impact that the expression of β-ARs might have on NF-kB/COX-2 signalling activation when taxane-based chemotherapy is planned and further confirmed the role of β-ARs in driving resistance to standard chemotherapy. To expand the range of sarcomas examined, we conducted a pilot study on the targeting of β-ARs with propranolol in an SFT patient and in patient-derived cells, further confirming the functional role of β-ARs in the proliferation and chemoresistance of sarcomas. Remarkably, the quantification of β-ARs in patient-derived cells and tissues highlighted that of the three β-AR receptors, the β2 subtype was strongly expressed in SFT and liposarcoma cells, which responded better to the combination of propranolol with docetaxel. Therefore, we can speculate that in such tumours, the β2-AR expression level might be indicative of tumour responsiveness to such chemotherapy. In conclusion, although propranolol was effective at high concentrations, it is worthwhile to consider that unlike continuous use, such as in patients^43^, propranolol was given as a single administration in our experiments and for that it was necessary to use high doses*.* Perhaps, the development of a non-competitive antagonist may achieve an irreversible inactivation of receptors at lower doses; however the toxicity of propranolol is well described on humans as well as its pharmacokinetic and this suggests to explore propranolol in different scenarios. Hence, overall our results support the repositioning of propranolol as an anticancer agent in STS and suggest a possible predictive role of β-ARs in the response to docetaxel and doxorubicin-based chemotherapy, which should be evaluated through well-designed clinical trials.

## Materials and methods

### Establishment of SFT short-term culture and AS cell line

Within the study SELDI, approved by the ethics committee of the IRCCS Instituto Tumori “Giovanni Paolo II” (n. 515/CE), after the signature of the written informed consent, specimens from either the patient with SFT and from that with AS were collected for the preparation of biopsy-derived tumor cells. The study was performed in accordance with the relevant guidelines and regulations. The fragments from both lesions were provided by the Institutional Histopathological Unit and processed as described in ^22^ to obtain a short-term culture of STF and the angiosarcoma cell line, designated AS-O2. The second was routinely monitored for mycoplasma contamination and it is still proliferating.

### Cells and culture conditions

The human LMS cell line SK-LMS-1 (ATCC HTB-88) was cultured in Eagle’s Minimum Essential Medium (EMEM, ATCC 30-2003); the human LPS cell line SW-872 (ATCC HTB-92) was cultured in Leibovitz's L-15 Medium (ATCC 30-2008), whereas the patient-derived SFT short-term culture and AS cell line were cultured in DMEM. All cell culture mediums were supplemented with 100 U/ml penicillin, 100 µg/mL streptomycinand 10% (v/v) heat-inactivated FBS (Gibco BRL, Life Technology, Breda, The Netherlands), with the only exception of DMEM in which 2 mM glutamine was added. All cells were grown at 37 °C in a humidified atmosphere with 5% CO_2_.

### Immunohistochemistry (IHC)

Paraffin sections (4 μm) of formalin-fixed tissue, obtained from surgical samples of LMS, LPS and AS patients were provided by the Histopathological Unit according to the study (n. 515/CE). The study was performed in accordance with the relevant guidelines and regulations. The tissue sections were deparaffinized, rehydrated, and processed for antigen retrieval with Sodium Citrate Buffer (10 mM Sodium Citrate, 0.05% Tween 20, pH 6.0) in a thermostatic bath at 98 °C for 30 min or alternatively with 0.1% trypsin in PBS at room temperature (RT) for 30 min, according to primary antibody manufacturer’s protocol. To avoid nonspecific binding, the sections were incubated in 5% FBS in PBS at RT for 1 h. Primary antibodies were: anti-β1 Adrenergic Receptor (Abcam), anti-β2 Adrenergic Receptor (Sigma) and anti-β3 Adrenergic Receptor (Abcam) in 5% FBS in PBS, incubated overnight at 4 °C. Thereafter the sections were washed in PBS twice and treated with 3% hydrogen peroxide for 15 min to quench endogenous peroxidase. After 3 wash steps with PBS, the sections were incubated with Envision + System-HRP Labelled polymer secondary antibody (Dako, Denmark; anti-rabbit) or the secondary antibody Goat anti-chicken IgY-HRP (Abcam) in PBS with 5% FBS for 1 h. The sections were then developed with Liquid DAB + Substrate Chromogen System or AEC + High Sensitivity Substrate Chromogen ready-to-use (Dako, Denimark), counterstained with hematoxylin and sealed with Kaiser mounting medium for optical observation by means of light microscopy.

### Immunocytochemistry (ICC)

Cells were seeded into glass Lab-Tek Chamber Slides (8 wells; 0.8cm2/well) at a density of 15–20 × 10^3^/well, allowed to attach for 24 h at 37 °C. For P-gp detection AS cells were previously treated with 1 µg/ml docetaxel, 150 µM propranolol and their combination. Once the confluence was reached the cells were fixed for 10 min in acetone at RT and treated with a blocking solution. Then, the slides were incubated with diluted primary antibody anti-β1 Adrenergic Receptor (Abcam), anti-β2 Adrenergic Receptor (Abcam), anti-β3 Adrenergic Receptor (Abcam) and anti-P-gp (JSB-1, Abcam) in 1% BSA in PBS for 1 h at RT. After 3 wash steps with PBS, cells were then incubated with EnVision + System-HRP Labelled polymer secondary antibody (Dako, Denmark). Color development was obtained with AEC ready to use solution (Dako, Denmark), whereas the nuclei were counterstained with hematoxylin. Then, the slides were sealed with Kaiser mounting medium for optical observation.

For the evaluation of CD-31 and CD-34,the slides were incubated with diluted primary antibody anti-CD-31 (PECAM-1, Novocastra) and anti-CD-34 (clone QBEND-10, Novocastra) in 3% BSA in PBS for 1 h and revealed by secondary antibody FITC-Goat anti-mouse Ig (BD Biosciences). Thereafter the slides were sealed with Vectashield (Vector Laboratories) for fluorescence examination by means of DMi8 Leica microscope.

### In vitro capillary morphogenesis assay

Matrigel (50 µl; 10–12 mg/ml) was pipetted into 96-well plates and polymerized for 30 min to 1 h at 37 °C, as previously described^44^. AS cells were seeded at a density of 12 × 10^3^ cells/well. The growth, morphogenesis and organization of AS cells were recorded after 3 h and 6 h with an inverted microscope (Olympus CKX41) equipped with a digital camera and an analysis system (LCmicro).

### Drugs and chemicals

Doxorubicin and propranolol were purchased from Selleckchem. Stock solutions were prepared at 10 mM and 50 mM in dimethylsulfoxide (DMSO) and stored in aliquots at − 20 °C and − 80 °C, respectively. The chemotherapeutic drugs Vinorelbine (Vinorelbine Injection 50 mg/5 ml-Sandoz) and Docetaxel (Docetaxel Accord 160 mg/8 ml-Accord) were supplied by the Institutional Pharmacy Unit. Further dilutions were made in culture medium.

### Cell growth Inhibition studies

Cell growth inhibition was determined by using the 3-[4,5-dimethylthiazol-2-yl]-2,5-diphenyltetrazoliumbromide (MTT) assay. A total of 5 × 10^3^ cells/well were seeded in 96-well plates and allowed to attach for 24 h, then the cells were exposed to various concentrations of drug for 24 or 72 h. The LMS and LPS cell lines were treated with propranolol in a range of concentration between 25 and 150 µM. Doxorubicin was given in the range 1–50 µM, vinorelbine 1–50 ng/ml and docetaxel in the range 7.4–7,400 ng/ml in both cell lines. The AS cells were treated with 0.01 and 1 µg/ml docetaxel or 100 µM propranolol, instead the SFT cells with 1 µg/ml docetaxel and 100 µM propranolol. In each plate, one column contained cells which were exposed to vehicle (untreated cells), while each concentration of drug(s) was repeated in 6 identical wells. Results are expressed as log dose–effect curves reporting the fraction of unaffected (surviving) cells versus drug concentration. The combination index (CI) was calculated as described in ^45^. Each experiment was performed in triplicates.

### P-gp membrane expression

SK-LMS-1 and SW-872 cells were treated with 10 µM doxorubicin, 100/150 µM propranolol and their combination. After 4 and 24 h of treatment, the cells were harvested, washed in PBS1X and resuspended in 3% BSA in PBS. To evaluate P-gp membrane expression, the cells were incubated for 30 min at 4 °C with monoclonal anti-P-gp (JSB-1, Abcam) in 3% BSA in PBS. To determine the unspecific fluorescence, due to the fluorescein-conjugated secondary antibody, the cells were incubated with the isotype control (Purified Mouse IgG1 k isotype control, BD Pharmingen). After two wash steps with PBS, the cell pellet was resuspended in 3% BSA in PBS and incubated with FITC Goat anti-mouse Ig secondary antibody (BD Pharmingen) for 30 min at 4 °C. P-gp protein expression on cell membrane was determined by using a FACScan flow cytometer and the data analysis was carried out with the CellQuest software (Becton Dickinson, NJ).

### Intracellular doxorubicin accumulation

SK-LMS-1 and SW-872 cells were seeded in 30 mm diameter dishes at a density of 3 × 10^5^ cells/dish. Cells were allowed to attach for 24 h at 37ºC and then treated with 10 µM doxorubicin alone and in combination with 100/150 µM propranolol for 30 min, 4 h and 24 h. Thereafter the cells were harvested, washed twice in PBS (pH 7.4) and subjected to fluorescence analysis using FACScan flow-cytometeras previously described^46^ when using a fluorescent drug. The data analysis was carried out with the CellQuest software (Becton Dickinson, NJ)^47^.

### Western blot analysis

SK-LMS-1 and SW-872 cells were treated with 10 µM doxorubicin and 150 and 100 µM propranolol, respectively, and their combination, while, AS cells were treated with 1 µg/ml docetaxel and 100 µM propranolol alone and in combination. After 24 h, the protein extracts of cells, untreated and treated, were obtained and analyzed as described in ^48^. In each lane of a Bio-Rad minigel system (Bio-Rad), 40 μg protein were loaded, resolved by electrophoresis, and transferred to a PVDF membrane (Millipore). PVDF membranes were blocked for 1 h at RT in blocking buffer (5% BSA in TBS-Tween 20 0,1%) and incubated overnight at 4ºC with specific primary antibodies against p-Akt and Akt (rabbit polyclonal; Cell Signaling Technology), p- Erk1/2 (mouse monoclonal; Cell Signaling Technology), Erk1/2 (rabbit polyclonal; Cell Signaling Technology), COX-2 (mouse monoclonal; Cayman CHEMICAL) and NFkB p65 (rabbit monoclonal; Cell Signaling Technology). A rabbit-HRP and a mouse-HRP (Bio-Rad Laboratories, USA) were used as secondary antibody. The blot detection was performed with ChemiDoc Imaging Systems and analyzed with the ImageLab software (Bio-Rad-USA). β-actin expression levels were used to normalize the sample values.

### Statistics analysis

All in vitro experiments were performed in triplicate, and results have been expressed as the mean ± standard deviation (SD) unless otherwise indicated. Statistical differences of in vitro data were assessed by the Student–Newman–Keuls test and a two-way analysis of variance (ANOVA) followed by Bonferroni post tests (GraphPad Prism ver. 5). The data were considered significant for *p* values (*) lower than 0.05(*), 0.01 (**) and 0.001 (***).

## Supplementary information

Supplementary information

## References

[1] Cloutier JM, Charville GW (2019). Diagnostic classification of soft tissue malignancies: a review and update from a surgical pathology perspective. Curr. Probl. Cancer.

[2] Walczak BE, Irwin RB (2013). Sarcoma chemotherapy. J. Am. Acad. Orthop. Surg..

[3] Riedel RF (2012). Systemic therapy for advanced soft tissue sarcomas: highlighting novel therapies and treatment approaches. Cancer.

[4] Le Cesne A (2014). Doxorubicin-based adjuvant chemotherapy in soft tissue sarcoma: pooled analysis of two STBSG-EORTC phase III clinical trials. Ann. Oncol..

[5] Mora J (2011). Advances and controversies in the management of high-risk neuroblastoma. Pediatr. Catalana.

[6] Di Martile M (2018). Histone deacetylase inhibitor ITF2357 leads to apoptosis and enhances doxorubicin cytotoxicity in preclinical models of human sarcoma. Oncogenesis.

[7] Meyer M, Seetharam M (2019). First-line therapy for metastatic soft tissue sarcoma. Curr. Treat. Options Oncol..

[8] Mangoni M (2019). Soft tissue sarcomas: new opportunity of treatment with PARP inhibitors?. Radiol. Medica.

[9] Pollack SM, Ingham M, Spraker MB, Schwartz GK (2018). Emerging targeted and immune-based therapies in sarcoma. J. Clin. Oncol..

[10] Borgatti A (2017). Safe and effective sarcoma therapy through bispecific targeting of EGFR and uPAR. Mol. Cancer Ther..

[11] Hasegawa H, Saiki I (2002). Psychosocial stress augments tumor development through β-adrenergic activation in mice. Jpn. J. Cancer Res..

[12] Veksler IG, Riabukha VN, Smelkova MI, Balitskiĭ KP (1984). Changes in the metastasis of experimental tumors and in the antimetastatic effect of cytostatics in their pharmacological action on adrenergic processes. Eksp. Onkol..

[13] Palm D (2006). The norepinephrine-driven metastasis development of PC-3 human prostate cancer cells in BALB/c nude mice is inhibited by β-blockers. Int. J. Cancer.

[14] Rains SL, Amaya CN, Bryan BA (2017). Beta-adrenergic receptors are expressed across diverse cancers. Oncoscience.

[15] Hieble JP, Bondinell WE, Ruffolo RR (1995). α- and β-adrenoceptors: from the gene to the clinic. 1. Molecular biology and adrenoceptor subclassification. J. Med. Chem..

[16] Lefkowitz RJ (2007). Seven transmembrane receptors: something old, something new. Acta Physiol..

[17] Cole SW, Sood AK (2012). Molecular pathways: beta-adrenergic signaling in cancer. Clin. Cancer Res..

[18] Schuller HM (2017). A new twist to neurotransmitter receptors and cancer. J. Cancer Metastasis Treat..

[19] Wessler I, Kirkpatrick CJ (2008). Acetylcholine beyond neurons: The non-neuronal cholinergic system in humans. Br. J. Pharmacol..

[20] Chisholm KM (2012). β-Adrenergic receptor expression in vascular tumors. Mod. Pathol..

[21] Stiles JM (2013). Targeting of beta adrenergic receptors results in therapeutic efficacy against models of hemangioendothelioma and angiosarcoma. PLoS ONE.

[22] Azzariti A (2014). Irradiation-induced angiosarcoma and anti-angiogenic therapy: a therapeutic hope?. Exp. Cell Res..

[23] Shaheen NL (2017). Extracellular matrix composition modulates angiosarcoma cell attachment and proliferation. Oncoscience.

[24] Brohée L (2018). Propranolol sensitizes prostate cancer cells to glucose metabolism inhibition and prevents cancer progression. Sci. Rep..

[25] Sun B (2018). Propranolol inhibits proliferation and invasion of hemangioma-derived endothelial cells by suppressing the DLL4/Notch1/Akt pathway. Chem. Biol. Interact..

[26] Veliz M, Chang V, Kasimis B, Choe JK (2007). Durable response of angiosarcoma of the face and scalp to docetaxel. Clin. Oncol..

[27] Yamada M, Hatta N, Mizuno M, Oishi N, Takehara K (2005). Weekly low-dose docetaxel in the treatment of lung metastases from angiosarcoma of the head [7]. Br. J. Dermatol..

[28] Penel N (2008). Phase II trial of weekly paclitaxel for unresectable angiosarcoma: the ANGIOTAX study. J. Clin. Oncol..

[29] Niso M (2013). Sigma-2 receptor agonists as possible antitumor agents in resistant tumors: hints for collateral sensitivity. ChemMedChem.

[30] Porcelli L, Lemos C, Peters G, Paradiso A, Azzariti A (2009). Intracellular trafficking of MDR transporters and relevance of SNPs. Curr. Top. Med. Chem..

[31] Bachmakov I, Werner U, Endress B, Auge D, Fromm MF (2006). Characterization of β-adrenoceptor antagonists as substrates and inhibitors of the drug transporter P-glycoprotein. Fundam. Clin. Pharmacol..

[32] Liu S (2017). PD-1/PD-L1 interaction up-regulates MDR1/P-gp expression in breast cancer cells via PI3K/AKT and MAPK/ERK pathways. Oncotarget.

[33] Li QQ (2007). Involvement of CD147 in regulation of multidrug resistance to P-gp substrate drugs and in vitro invasion in breast cancer cells. Cancer Sci..

[34] Shen H (2011). Upregulation of mdr1 gene is related to activation of the MAPK/ERK signal transduction pathway and YB-1 nuclear translocation in B-cell lymphoma. Exp. Hematol..

[35] Xie X, Tang B, Zhou J, Gao Q, Zhang P (2013). Inhibition of the PI3K/Akt pathway increases the chemosensitivity of gastric cancer to vincristine. Oncol. Rep..

[36] García MG, Alaniz LD, Cordo Russo RI, Alvarez E, Hajos SE (2009). PI3K/Akt inhibition modulates multidrug resistance and activates NF-κB in murine lymphoma cell lines. Leuk. Res..

[37] Alemany R (2018). Nilotinib as coadjuvant treatment with doxorubicin in patients with sarcomas: a phase I trial of the spanish group for research on sarcoma. Clin. Cancer Res..

[38] Subbaramaiah K, Marmo TP, Dixon DA, Dannenberg AJ (2003). Regulation of cyclooxgenase-2 mRNA stability by taxanes: evidence for involvement of p38, MAPKAPK-2, and HuR. J. Biol. Chem..

[39] Singh B (2008). Cyclooxygenase-2 induces genomic instability, BCL2 expression, doxorubicin resistance, and altered cancer-initiating cell phenotype in MCF7 breast cancer cells. J. Surg. Res..

[40] Tomita Y (2006). Prognostic significance of activated AKT expression in soft-tissue sarcoma. Clin. Cancer Res..

[41] Steelman LS (2011). Involvement of Akt and mTOR in chemotherapeutic- and hormonal-based drug resistance and response to radiation in breast cancer cells. Cell Cycle.

[42] Liu J, Mao W, Ding B, Liang C (2008). ERKs/p53 signal transduction pathway is involved in doxorubicin-induced apoptosis in H9c2 cells and cardiomyocytes. Am. J. Physiol. Circ. Physiol..

[43] Hajighasemi F (2009). In vitro sensitivity of leukemia cells to propranolol. J. Clin. Med. Res..

[44] Serratì S (2009). TGFβ1 antagonistic peptides inhibit TGFβ1-dependent angiogenesis. Biochem. Pharmacol..

[45] Chou T-C (2010). Drug combination studies and their synergy quantification using the Chou–Talalay method. Cancer Res..

[46] Iacobazzi RM (2017). Targeting human liver cancer cells with lactobionic acid-G(4)-PAMAM-FITC sorafenib loaded dendrimers. Int. J. Pharm..

[47] Depalo N (2017). Sorafenib delivery nanoplatform based on superparamagnetic iron oxide nanoparticles magnetically targets hepatocellular carcinoma. Nano Res..

[48] Porcelli L (2015). Aurora kinase B inhibition reduces the proliferation of metastatic melanoma cells and enhances the response to chemotherapy. J. Transl. Med..

