# Supplementary material for: The β-adrenergic receptor antagonist propranolol offsets resistance mechanisms to chemotherapeutics in diverse sarcoma subtypes: a pilot study
Source: Sci Rep. 2020 Jun 26;10:10465. doi: 10.1038/s41598-020-67342-6 (PMC7320177; doi:10.1038/s41598-020-67342-6)
Supplement: Supplementary file 1 — Supplementary information [file 41598_2020_67342_MOESM1_ESM.pdf]

β-adrenergic receptors antagonist propranolol offset resistance mechanisms to chemotherapeutics in diverse sarcoma subtypes: a pilot study

Letizia Porcelli[1,#], Marianna Garofoli[1,,#], Roberta Di Fonte[1], Livia Fucci[3], Mariateresa Volpicella[2], Sabino Strippoli[4], Michele Guida[5]and Amalia Azzariti[1]*

[1]Experimental Pharmacology Laboratory, [3]Histopathological Unit, [5]Medical Oncology, Istituto Tumori Giovanni Paolo II, Bari, Italy. [2]Department of Biosciences, Biotechnologies and Biopharmaceutics, University of Bari, Bari, Italy.[4]Medical Oncology Unit, Ospedale Mons. R. Dimiccoli Barletta, Bat, Italy
# equally contributed, * corresponding author

*****Corresponding author
Amalia Azzariti, PhD
Experimental Pharmacology Laboratory
Istituto Tumori Giovanni Paolo II
Viale O. Flacco, 65 - 70124 Bari, ITALY
Phone/Fax: +39-080-5555986
E-mail: a.azzariti@oncologico.bari.it

LEIOMIOSARCOMA IMMUNOHISTOCHEMISTRY

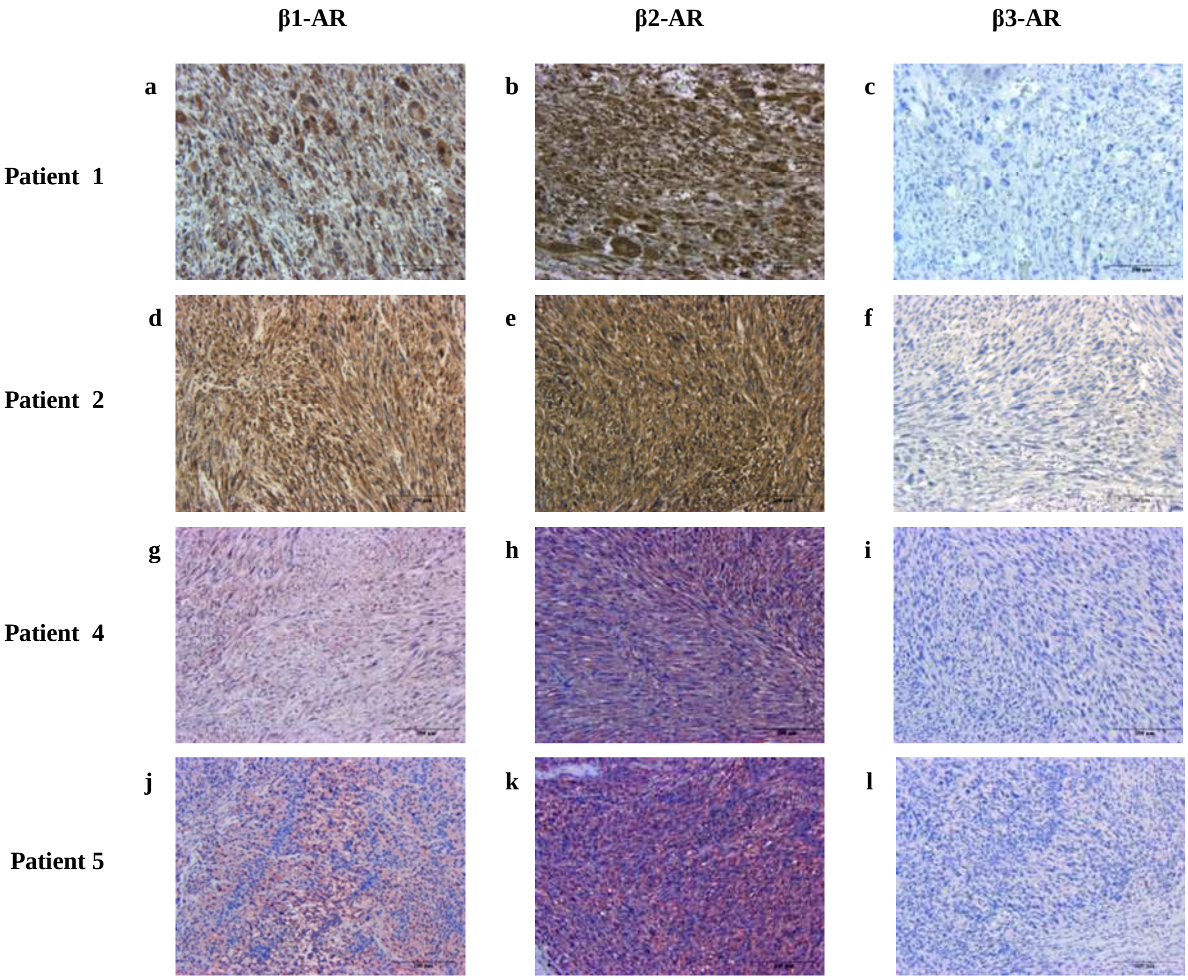

Supplementary Figure S1: Immunohistochemical evaluation of β1-AR, β 2-AR, and β3-AR in leiomyosarcoma tissues specimens. Images scale bar 200µm.

LIPOSARCOMA IMMUNOHISTOCHEMISTRY

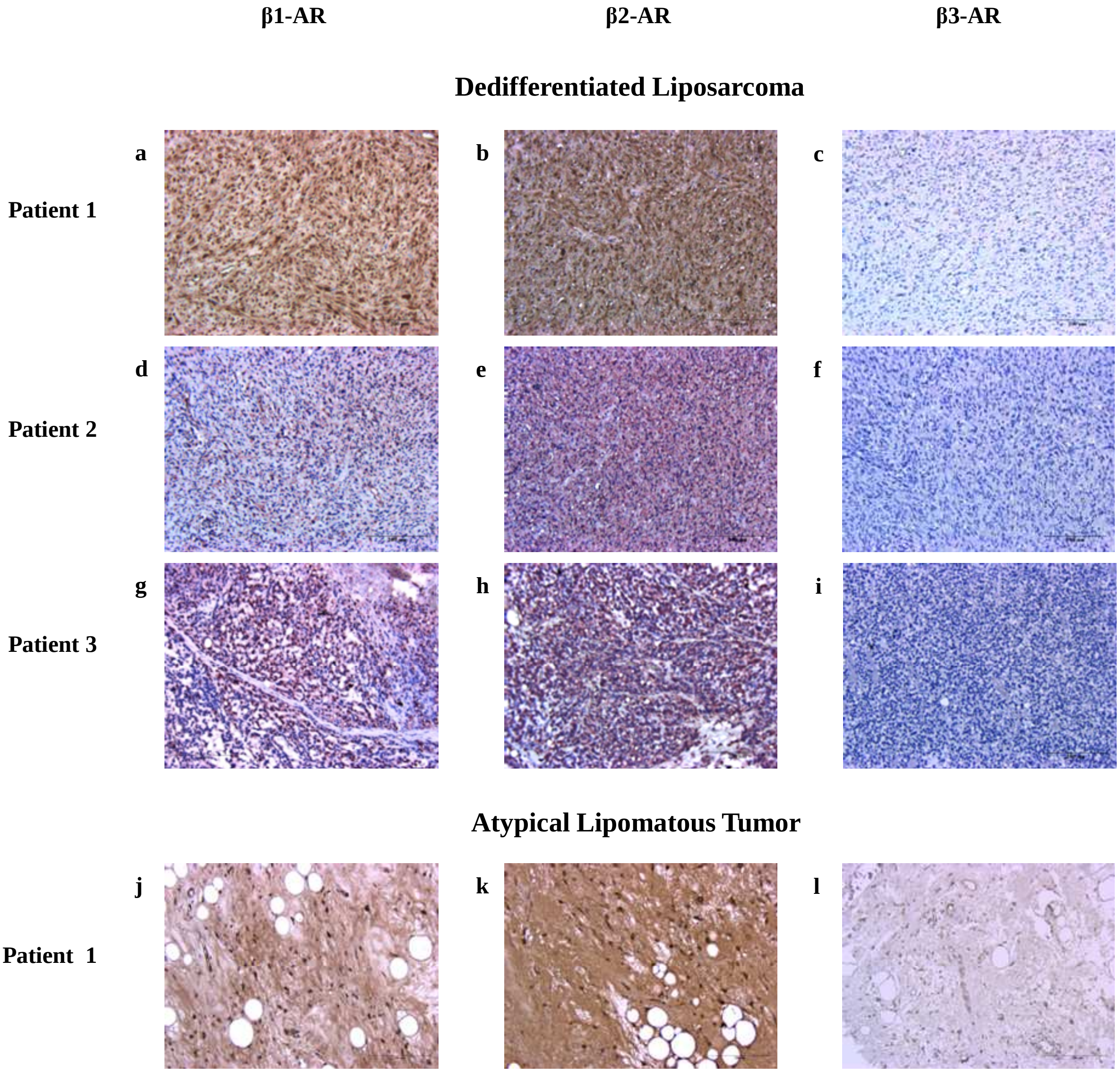

Supplementary Figure S2: Immunohistochemical evaluation of β1-AR, β 2-AR, and β3-AR in liposarcoma tissues specimens. Images scale bar 200µm.

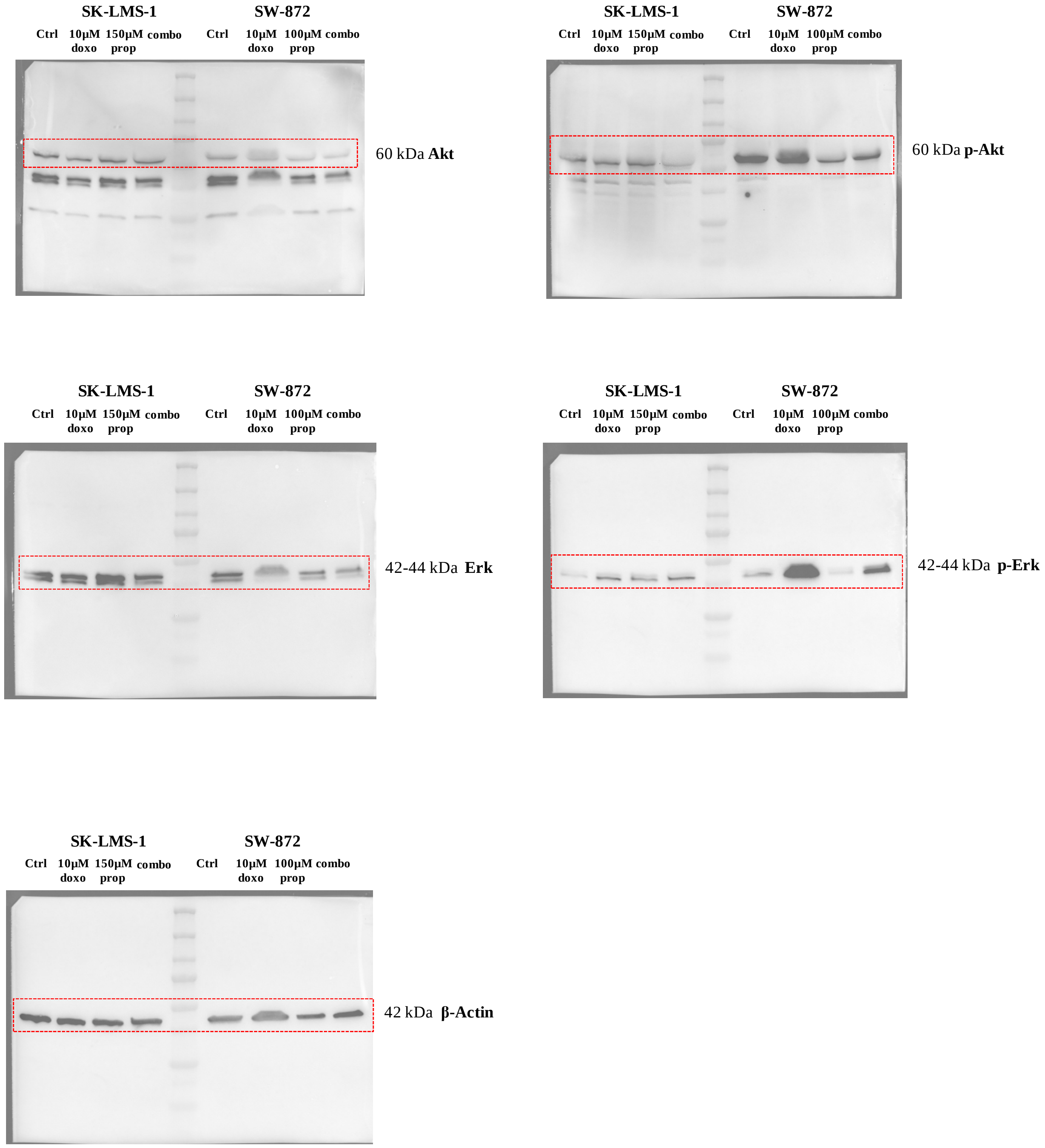

Supplementary FigureS3:Immunoblots performed on proteins extract from SK-LMS-1 and SW-872 cells treated with doxorubicin and propranolol, respectively, and their combination.

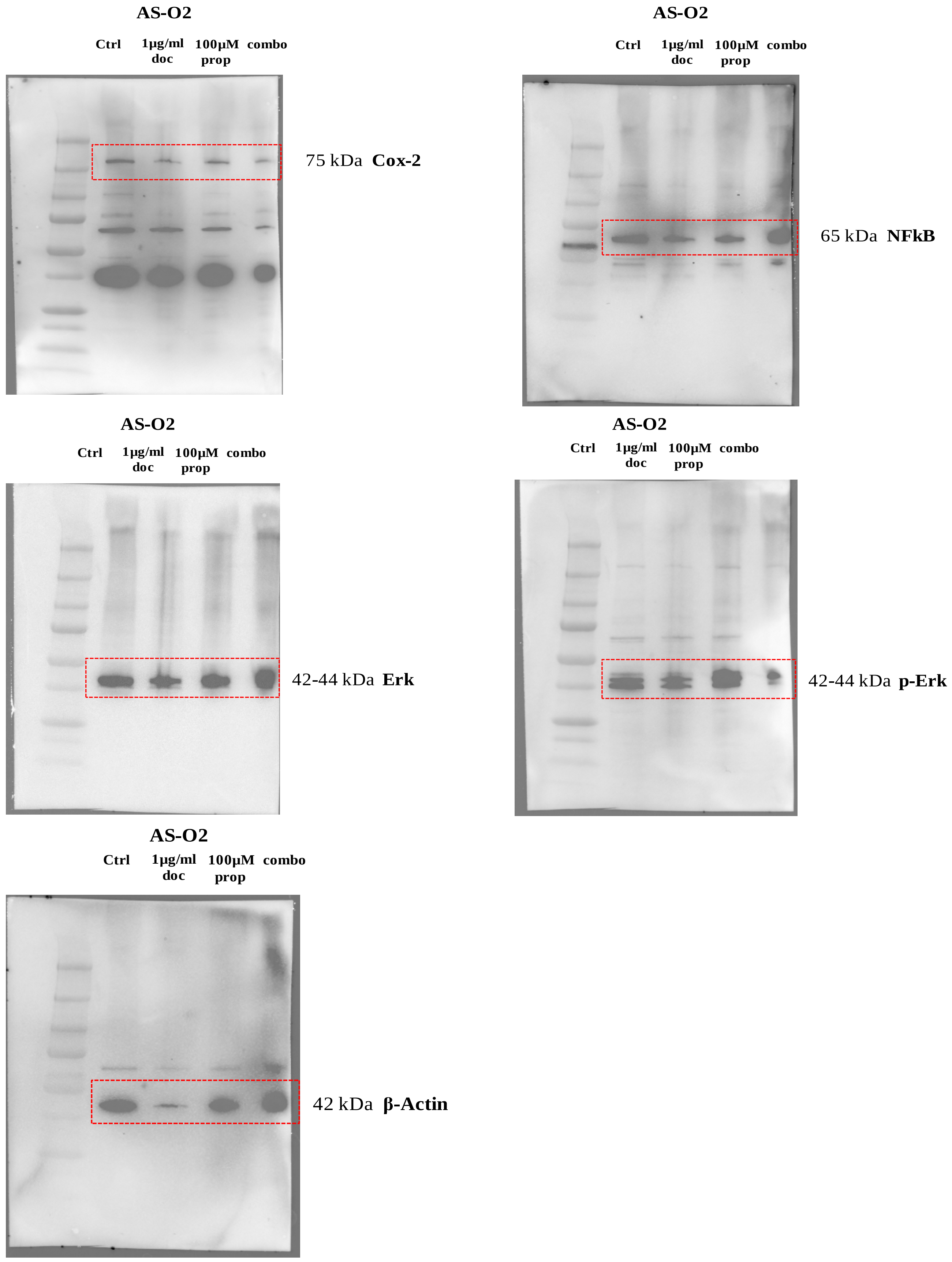

Supplementary Figure S4: Immunoblots performed on proteins extract from AS-O2 cells treated with dcetaxel and propranolol, respectively, and their combination.
